# Variability and validity of intimate partner violence reporting by couples in Tanzania

**DOI:** 10.1371/journal.pone.0193253

**Published:** 2018-03-08

**Authors:** Nafisa Halim, Ester Steven, Naomi Reich, Lilian Badi, Lisa Messersmith

**Affiliations:** 1 Department of Global Health, Boston University School of Public Health, Boston, MA, United States of America; 2 Muhimbili University of Health and Allied Sciences, Dar es Salaam, Tanzania; 3 World Education Inc./Bantwana Initiative, Boston, MA, United States of America; 4 World Education, Inc./Bantwana Initiative–Tanzania, Arusha, Tanzania; Stellenbosch University, SOUTH AFRICA

## Abstract

In recent years, major global institutions have amplified their efforts to address intimate partner violence (IPV) against women—a global health and human rights violation affecting 15–71% of reproductive aged women over their lifetimes. Still, some scholars remain concerned about the validity of instruments used for IPV assessment in population-based studies. In this paper, we conducted two validation analyses using novel data from 450 women-men dyads across nine villages in Northern Tanzania. First, we examined the level of inter-partner agreement in reporting of men’s physical, sexual, emotional and economic IPV against women in the last three and twelve months prior to the survey, ever in the relationship, and during pregnancy. Second, we conducted a convergent validity analysis to compare the relative efficacy of men’s self-reports of perpetration and women’s of victimization as a valid indicator of IPV against Tanzanian women using logistic regression models with village-level clustered errors. We found that, for every violence type across the recall periods of the last three months, the last twelve months and ever in the relationship, at least one in three couples disagreed about IPV occurrences in the relationship. Couples’ agreement about physical, sexual and economic IPV during pregnancy was high with 86–93% of couples reporting concordantly. Also, men’s self-reported perpetration had statistically significant associations with at least as many validated risk factors as had women’s self-reported victimization. This finding suggests that men’s self-reports are at least as valid as women’s as an indicator of IPV against women in Northern Tanzania. We recommend more validation studies are conducted in low-income countries, and that data on relationship factors affecting IPV reports and reporting are made available along with data on IPV occurrences. Keywords: Intimate partner violence; measurement; validity; survey research; Tanzania.

## I. Introduction

Intimate partner violence (IPV) is a global health and human rights problem affecting 15–71% of reproductive-aged women worldwide over their lifetime[1]. Sub-Saharan (30–66%) has one of the highest lifetime physical or sexual IPV rates globally, along with South Asia (42%) [2]. Defined as the threatened, attempted, and completed physical, sexual or psychological abuse that occurs between intimate partners, IPV is one of the most common forms of violence experienced by women across all societies and social hierarchies [3, 4]. IPV is associated with poor physical, psychological, and reproductive health outcomes, and productivity loss among women and poor health and developmental outcomes among children born to IPV-victimized women [1, 5, 6]. In extreme cases, IPV can result in death: 39% of all female homicides worldwide are the result of IPV [7].

Recently, IPV has evolved into an important area of intervention and investigation among practitioners and academics. Major global health and development organizations have escalated their commitment to address IPV in low-income countries. For example, since 2013, the United States’ (USAID and the Department of State) support for global gender-based violence (GBV) programs totaled approximately $153 million per year [8]. During this time, the WHO has provided healthcare professionals worldwide with technical assistance to build their capacity for IPV screening and response [9]. Currently, the World Bank supports $128 million in development projects aimed at addressing violence against women [10]. In addition, efforts to expand IPV evidence-base are underway. For example, since 1990, the Demographic and Health Surveys (DHS) have collected data on lifetime victimization from reproductive-aged women in 25 low- and middle-income countries across Africa, Asia, Latin America, the Middle East, and Eastern Europe [11].

However, some scholars remain concerned about the validity of instruments used for IPV assessment, questioning whether IPV instruments are effective in accurately classifying women as exposed or not exposed to IPV [12–16]. Data on IPV occurrences are typically captured using retrospective self-reports by the victim or the perpetrator on a battery of questions, each on an act of violence during a recall period. Thereby, self-reports can generate underreporting if victims or perpetrators conceal victimization or perpetration, misunderstand questions, or forget about IPV occurrences [13]. While validation studies remain scant in low-income countries (see [11] for exception), those in high-income countries confirmed this concern. Using self-reports by the victim *and* the perpetrator, scholars showed poor inter-partner agreement in IPV reporting. Regardless of gender, victims report up to 50% more IPV occurrences than perpetrators, depending on context [17–20].

In this paper, we conducted two validation analyses. First, we examined the level of inter-partner agreement in the reporting of physical, sexual, emotional and economic IPV against women in the last three and twelve months prior to the survey, ever in the relationship, or during pregnancy, using novel data from 450 couples across nine villages in Northern Tanzania. Second, we conducted a convergent validity analysis to compare the relative efficacy of men’s self-reported perpetration and women’s self-reported victimization as a valid indicator of IPV against women in Tanzania. Using logistic regression models, we examined whether and to what extent male and female partners’ IPV self-reports were associated with men’s self-reports on validated risk IPV factors, namely, men’s inequitable attitudes about gender norms and relations, traumatic childhood experience, condom non-use, multiple sexual partners, and substance use.

This study makes important contributions to IPV research in low-income countries. First, since self-reports will dominate IPV research for years to come [21], rates of partner disagreement in IPV reporting will help obtain an estimate of prevalence of possible misreports. Also, they may inform calculation of “correction coefficients” to be adjusted for more accurate IPV prevalence estimates when using IPV self-reports by one partner. Second, convergent validity findings will show the relative performance of men’s vs. women’s self-reports for assessing IPV against women in Tanzania. Such validation is imperative for better understanding IPV, especially, in the context of poor inter-partner agreement.

## II. Setting

Gender-based stratification is a central feature of society in Tanzania, an East African country with an estimated population of 47.4 million and per capita income of $865 [22]. With a score of 0.547 on the Gender Inequality Index, Tanzania ranks 124 out of the 155 countries scored, indicating that disparities exist between Tanzanian men and women in economic status, empowerment, and reproductive health. Underpinning the gender-based stratification is a patriarchal system including patrilineal inheritance that deprives women of critical rights and privileges [23]. In Tanzania, the total fertility rate is 5.3; the adolescent fertility rate is 128.7 births per 1000 women ages 15–19; the maternal mortality ratio is 578 per 100,000; female secondary school enrollment rate is 24%; and land ownership among women of reproductive age is 5% [22]. Patriarchal traditions and institutions govern marriage, childbearing, age-disparate sexual relations, and sexual practices [24, 25]. For example, social pressures for early childbearing discourages condom use [26]; male dominance of sexual decision-making is expected [27]; and bridewealth, a cash or in-kind payment made by the man’s family to the woman’s, restricts women’s ability to negotiate safe sex or to deny sex [28].

## III. Factors affecting couples’ discordant IPV reporting

Discordant reporting of IPV by couples occurs when (a) the man reports no violence perpetration, but the woman reports victimization (Table 1: Cell 2); and (b) the man reports violence perpetration, but the woman reports no victimization (Table 1: Cell 3).

**Table 1:** Concordance and discordance in couples' IPV reporting.

		Male partners’ reports of:	
		Perpetration	No perpetration
Female partners’ reports of:	Victimization	(1)	(2)
		Concordance	Discordance
	No victimization	(3)	(4)
		Discordance	Concordance

Scholars attribute couples’ discordant reporting to a number of social and methodological factors. Social factors include social desirability and norms of victim blaming [29, 30]. Methodological factors include definitional differences and data collection methods and procedures [31].

Social desirability is often considered a determinant of couples’ discordant reporting and perpetrators’ under-reporting of IPV [3, 21]. Defined as the practice of reporting what is perceived as socially appropriate and acceptable, rather than what is a fact [32], social desirability poses one of the greatest threats to the validity of self-reported IPV data [33]. This is because, with questions inquiring about sensitive or antinormative behaviors or beliefs, researchers essentially ask individuals to disclose violations of social norms [34]. Individuals are likely to distort facts regarding their engagement in antinormative behaviors, since they value being viewed favorably by others, and positive social images [35]. Therefore, social desirability may encourage men with a history of violence perpetration not to disclose their perpetration behaviors, and women with a history of victimization to disclose more frequently than men their victimization experiences, where men-to-women IPV is inappropriate and stigmatized.

In Tanzania, social desirability will likely produce couples’ discordant men-to-women IPV reporting in the form portrayed in Cell 3 more commonly than that in Cell 2 (Table 1). As stated before, Tanzanian society is patriarchal, giving rise to a pro-male gender hierarchy, where rights and privileges are unequally distributed between men and women [24, 25, 36]. In Tanzania, where IPV against women is accepted, men may disclose their perpetration behaviors fearing no negative sanctions (e.g., public ridicule, shaming). According to the 2015–16 Tanzania DHS, one in three men, and two in three women justify IPV against women [37]. A USAID report (2008) found that IPV against women is perceived as “normal” by women and men of all ages, class, and geographic regions [38]. Both men and women condone IPV as a “measure to correct women in specific situations” [38, 39]. Women perceive a “good beating” as a legitimate way to enforce and demonstrate men’s compliance with masculine ideals [40]. Moreover, the Law of Marriage Act (2002) does not recognize marital rape as a punishable offence, though it prohibits ‘corporal punishment’ against a wife. In this context, research has shown that women victims rarely seek help from formal sources [28].

Conversely, Tanzanian women may not disclose victimization fearing blame, shame, divorce, abandonment, and loss of the custody of children. McCleary-Sills and colleagues (2016) explain, “… at the core of this is a sense that women are at fault for any violence they experience because they have somehow provoked their partners into beating them” (p. 229)[28]. Women fear that IPV signals to society that women have failed in their role as a wife and mother, bringing shame to women and their families [28]. Women fear additional negative repercussions of reporting IPV, including divorce, abandonment, loss of custody of children, loss of support, and violence escalation [28].

On methodological factors, couples’ discordant IPV reporting may be attributable to definitional differences. A difficult construct to operationalize, IPV tends to be private (i.e., occurs behind closed doors and beyond public eye) and subjective (i.e., spatial and temporal variations exist as to whether an act is perceived as violent). Men’s knowledge about what is or is not IPV may be lower than women’s, creating discordant reporting of IPV. Such gender gaps may exist because men only recently started participating in violence prevention programs (e.g., USAID’s CHAMPION project), whereas women’s participation in such programs has been ongoing for many years [41].

Finally, couples’ discordant IPV reporting may stem from questionnaire design and implementation [31]. Men and women may differ in interpretation of and response to survey contents or scale items that are ambiguous. For example, for a question that makes reference to more than one act of IPV (e.g., kicked, dragged, beat, choked or burned), men and women may respond in reference to two different behaviors [31]. Second, compared to men, women may be more likely to underreport when the interviewer is a man [42] and privacy is lacking [43, 44]. Finally, while women and men alike may underreport if there are concerns that interviewers will be unable to preserve confidentially and anonymity of the data collected [14, 31], women may be more prone to underreporting anticipating violence escalation by men angered at women’s disclosure to interviewers of private family matters.

## IV. Risk factors for men’s perpetration of IPV against their partners

For convergent validation analysis, the IPV risk factors considered include men’s inequitable gender attitudes; exposure to childhood trauma; multiple sexual partners; condom non-use; and alcohol or drug use. Support in prior literature informed inclusion of these IPV risk factors.

Men’s excessive alcohol consumption is associated with IPV against women. Working as a trigger, alcohol influences violence by disinhibiting behavior in individuals with aggressive tendencies and encouraging sexual-risk taking (e.g., extramarital sex, high numbers of casual sex partners; engaging in transactional sex, inconsistent condom use), which may lead to jealousy, perceptions of partners’ infidelity, transmission of sexually transmitted infections, and marital conflict and violence [45, 46]. Men’s alcohol consumption has been shown to be a risk factor for physical or sexual violence among women in Tanzania [24, 47, 48], Uganda [46], South Africa [49], India [50–52], among other places. Having multiple sexual partners and inconsistent condom use were shown to be a risk factor for at least one form of violence in Tanzania [24, 48], South Africa [49], India [53], Bangladesh [54], Nepal [55].

Childhood exposure to traumatic experiences, especially when untreated, can influence individuals’ violent treatment of others later in life [56, 57]. Childhood exposure to violence, maltreatment and neglect may influence formative perceptions of what is “normal” in intimate relationships, reinforcing later life tendencies to normalize and perpetrate IPV [58–60]. Consistently, studies have found that childhood exposure to traumatic experiences is a risk factor for at least one form of violence against women in Tanzania [47, 48], South Africa [61], Malawi [62], Vietnam [63], and India [53].

Finally, men’s IPV perpetration is believed to be a consequence of the gender system that entitles men to more rights, privileges and power, resulting in gender inequalities at the societal and relational levels. This inequality limits women’s educational attainment and wage employment, leading to women’s economic dependence on men and vulnerability to and acceptance of male control and abuse of power [24, 64], and maintains women’s subordination in sexual relationships. Consistently, power inequity was found to be a risk factor for at least one form of violence in women in Tanzania [48] and South Africa [65].

## V. Data and measurements

For this analysis, we used the baseline survey data from 450 couples who participated in a three-arm cluster-randomized controlled trial (RCT) to evaluate the effectiveness of an intervention (entitled Together to End Violence against Women (TEVAW)) in reducing men’s IPV perpetration in Tanzania. We conducted this cluster-RCT in *Karatu* District, one of seven districts in the *Arusha* region of Tanzania. Our selection of this region and district was based on the high prevalence (>1 in 3) and acceptance (1 in 3) of IPV in *Arusha* (NBS & ICF Macro 2016), as well as the implementation of an ongoing savings groups (known as LIMCA) with women implemented by our partner, World Education Inc./Bantwana. These groups aimed to empower women participants through savings and credit activities to increase their economic independence and expand social networks. The groups also aimed to improve women’s knowledge about the physical, mental and emotional harms of IPV on women, men and children.

We recruited a non-probability sample of 450 of 604 women LIMCA members and their male partners. Women LIMCA members were briefed about the study during regular LIMCA meetings. We invited women-men dyads who were eligible. Eligibility criteria for women included being aged 18 years or above, living with a male partner, providing written consent to participate in the study and consents in writing to her male partner to participate. Eligibility criteria for men included being aged 18 year or above, living with a woman LIMCA group member, and providing written consent to participate in the study. We included in the study the first 450 men-women dyads deemed eligible. Baseline data were collected in June-July 2015 via face-to-face interviews. Women and men were interviewed separately in a private location by trained interviewers of the same gender. Ethical approval was obtained from Boston University and the Tanzania National Institute of Medical Research Institutional Review Boards.

### Measures

#### Dependent variables

For IPV by type, we adopted questions from three instruments: Men’s Health and Relationship Study [66], WHO Multi-Country Study on Women’s Health and Domestic Violence against Women [67], and the International Men and Gender Equality Study [68].

We created a dummy variable of whether a man (woman) had perpetrated (been victimized of) IPV against women. We considered that a man had perpetrated physical violence against his female partner in last three months, last 12 months, ever in the relationship, and during pregnancy if, for each of these time intervals, he reported perpetrating once, a few times or many times any one of the following: slapped a partner or thrown something at her that could hurt her; pushed or shoved a partner; hit a partner with a fist or with something else that could hurt her; kicked, dragged, beaten, choked or burned a partner; or threatened to use or actually used a gun, knife or other weapon against a partner. We considered that a woman had experienced physical violence from her male partner if she reported experiencing once, a few times or many times any one of these acts. The same parameters were used for men’s perpetration and women’s experience in the four time points mentioned of the following acts of sexual violence (forced a partner to have sexual intercourse when she did not want to; or forced a partner to do something sexual that she found degrading or humiliating), emotional violence (insulted a partner or deliberately made her feel bad about herself; belittled or humiliated a partner in front of other people; done things to scare or intimidate a partner on purpose; threatened to hurt a partner; or hurt people she cares about as a way of hurting her, or damaged things that are important to her), and economic violence (prohibited a partner from getting a job, going to work, trading or earning money; taken a partner’s earnings against her will; thrown a partner out of her house; or kept money from a partner’s earnings for alcohol, tobacco or other things knowing that partner was finding it hard to afford household expenses). Questions on physical, sexual, or economic IPV during pregnancy were about victimization of any acts of physical, sexual or economic IPV during any pregnancy.

#### Explanatory variables

Gender inequitable attitudes was measured using an 18-item scale on attitudes toward gender norms in intimate relationships, known as the GEM (Gender Equitable Men) Scale [69]. For each item listed, men reported their level of agreement with Strongly Agree = 4], Agree = 3], Disagree = 2], and Strongly Disagree = 1]. Men get a score ranging between 72 and 18, where 72 indicates the most inequitable gender attitudes on gender norms and relations and 18 indicates the least inequitable attitudes.

Exposure to childhood trauma was measured using a 13-item scale, a modified version of the Child Trauma Questionnaire [70]. For each item listed, men responded with Very Often = 4], Often = 3], Sometimes = 3], and Never = 1]. Men’s responses across all 13 items were added up allowing men to receive a score ranging between 52 and 13, where 52 indicates the most traumatic childhood and 13 indicates the least.

Condom non-use was measured using men’s reports on how often (never; occasionally; mostly; always) they used condoms when having sex in the last year. On condom non-use, men received the value of 1 if they reported never; else, they received a zero. Multiple sexual partners was measured using men’s reports on the question: “Including stable partners and occasional partners, how many people have you had sex with in the last year?” Options include: 1 person; 2 or 3 people; 4–10 people; 11–20 people; more than 20 people; and none. Men received 1 if they reported having sex with more than one person within the last year; otherwise, they received a zero. Finally, alcohol or drug use was measured using men’s reports on two questions: “How often do you drink alcohol?” and “How many times have you used drugs in the last 12 months?” For both questions, options include: every day or nearly every day; weekly; once a month; less than once a month; and never. Men received a zero if they reported never on both questions; otherwise, they received a one.

Finally, male and female partners’ age was measured with self-reported age in years; highest level of schooling attended was measured using self-reports.

## VI. Analysis

We estimated the crude percentage agreements and chance-corrected agreements between couples’ reports of physical, sexual, emotional, and economic violence in the last three and twelve months prior to the survey, ever in the relationship, and during pregnancy. We cross-tabulated men’s reports (Yes/No) of physical, sexual, emotional and economic violence perpetration in the last three and twelve months prior to the survey, ever in the relationship, and during pregnancy and women’s reports (Yes/No) of victimization. Further, we estimated the chance-corrected agreements between couples’ reports of each of the 48 violent events considered in the analysis (see Measures).

Next, for convergent validity analysis, we estimated the following equation:

$Y_{i}^{*}=\beta^{\prime }X_{i}+\mu_{i}$, where $Y_{i}^{*}$ is the log odds of reporting that IPV has occurred by man or woman *i. Y_i_* is 1 if the log odds of reporting IPV by men or women $Y_{i}^{*}$> 0. And, $\beta^{\prime }X_{i}=\beta_{0}+\sum_{k=1}^{K}\beta_{xk}X_{ki}+\beta_{M}M_{i}+\beta_{W}W_{i}$ and *X_i_* refers to risk factors for IPV perpetration, namely, men’s gender inequitable attitudes, exposure to childhood trauma, condom non-use, multiple sexual partners, and alcohol or drug use; *M_i_* refers to men’s age and highest level of education attained; and, *W_i_* refers to women’s age and highest level of education attained. We fit a logistic regression model with village-level clustered errors, controlling for male and female partners’ age and highest level of education attained.

## VII. Results

### Prevalence of concordant and discordant men-to-women IPV reporting

Table 2 presents couples’ crude (Columns 1 and 2) and chance-corrected (Column 7) agreement levels for reports on physical, sexual, emotional and economic IPV against women in the last three and twelve months prior to the survey, ever in the relationship, and during pregnancy (data on emotional IPV during pregnancy were not collected in this study).

**Table 2:** Percentage distribution of couples' cordordant and discordant reporting of four types of IPV in the last three or twelve months prior to the survey, ever in the relationship, or during pregnancy, Tanzania, 2015.

			(1)	(2)	(3)	(4)	(5)	(6)	(7)	
		*n*	Cordordant Reporting	Discordant Reporting	Man: *Yes*; Woman: *Yes*	Man: *Yes;* Woman: *No*	Man: *No;* Woman: *Yes*	Man: *No;* Woman: *No*	*Kappa*	*p*
Block 1: Last 3 Mos	Physical IPV	450	64	36	6	9	26	58	0.06	†
	Sexual IPV	450	60	40	5	6	34	55	0.04	
	Emotional IPV	450	52	48	18	10	38	34	0.09	*
	Economic IPV	450	61	39	10	15	24	51	0.07	†
Block 2: Last 12 Mos	Physical IPV	450	64	36	13	11	25	51	0.18	***
	Sexual IPV	450	59	41	7	6	35	52	0.06	†
	Emotional IPV	450	55	45	31	12	33	24	0.14	***
	Economic IPV	450	61	39	12	13	26	50	0.12	**
Block 3: Ever	Physical IPV	450	58	42	17	14	28	41	0.13	***
	Sexual IPV	450	53	47	8	7	40	45	0.02	
	Emotional IPV	450	56	44	39	13	31	17	0.10	**
	Economic IPV	450	60	40	18	13	27	42	0.17	***
Block 4: Pregnancy	Physical IPV	432	92	8	0	0	8	92	0.05	*
	Sexual IPV	436	93	7	0	3	4	93	0.08	*
	Economic IPV	446	86	14	1	2	13	85	0.05	†

***p≤.001

**p≤.01

*p≤.05

†p≤.10.

For every violence type across the recall periods of last three months, last twelve months and ever in the relationship, at least one in three couples disagreed about IPV occurrences in the relationship (Table 2; Blocks 1–3; Column 2). That is, the proportion of couples who disagreed about IPV occurrences ranges between 36% (physical IPV) and 48% (emotional IPV) for the recall period of last three months; between 36% (physical IPV) and 45% (emotional IPV) for the recall period of last twelve months; between 40% (economic IPV) and 47% (sexual IPV) for the recall period of ever in the relationship. Estimates of chance corrected agreement (*kappa*) between couples’ reports of IPV in last three months were low ranging between 0.04 (sexual IPV) and 0.09 (emotional IPV); *kappa* estimates for couples’ reports of IPV in the last twelve months and ever in the relationship were also low ranging between 0.06 (sexual IPV) and 0.18 (physical IPV) and between 0.02 (sexual IPV) and 0.17 (economic IPV), respectively.

As for patterns of disagreement in couples’ reports shown in Cells 2–3 of Table 1, couples whose female partners reported IPV victimization when male partners did not report IPV perpetration appeared in the sample up to five times as frequently as couples whose male partners reported IPV perpetration and female partners did not report any victimization (Table 2; Blocks 1–3; Columns 4–5). For example, the proportion of couples whose female partners reported victimization when male partners did not repot perpetration ranged between 24% (economic IPV) and 38% (emotional IPV) for last three months; between 25% (physical IPV) and 35% (sexual IPV) for last twelve months; and between 27% (economic IPV) and 40% (sexual IPV) for ever in the relationship (Table 2; Blocks 1–3; Columns 5). Conversely, the proportion of couples whose male partners reported IPV perpetration when female partners did not report victimization ranged between 6% (sexual IPV) and 15% (economic IPV) for last three months; between 6% (sexual IPV) and 13% (economic IPV) for last twelve months; and between 7% (sexual IPV) and 14% (physical IPV) (Table 2; Blocks 1–3; Columns 4).

Couples tend to disagree more about what form of IPV occurred in the relationship than they do about when that form of IPV occurred in the relationship. For instance, 36% vs. 48% of couples disagreed about physical vs. emotional IPV occurring in last three months (Table 2; Block 1; Column 2); 36% vs. 45% of couples disagreed about physical vs. emotional IPV occurring in last twelve months (Table 2; Block 2; Column 2); and 40% vs. 47% of couples disagreed about economic vs. sexual IPV occurring ever in the relationship (Table 2; Blocks 3; Column 2). Conversely, 36% vs. 42% of couples disagreed about physical IPV occurring in last three months vs. ever in the relationship (Table 2; Blocks 1–3; Column 2); 40% vs. 47% of couples disagreed about sexual IPV occurring in last three months vs. ever in the relationship (Table 2; Blocks 1–3; Column 2); 48% vs. 44% of couples disagreed about emotional IPV occurring in last three months vs. ever in the relationship (Table 2; Blocks 1–3; Column 2); and 39% vs. 40% of couples disagreed about economic IPV occurring in last three months vs. ever in the relationship (Table 2; Blocks 1–3; Column 2).

The proportion of couples disagreeing about IPV occurring during pregnancy is low ranging between 7% (sexual IPV) and 14% (economic IPV) (Table 2; Block 4; Column 2).

Further, we explored couples’ agreement when men reported that they had perpetrated violence and women reported that they had experienced violence (Columns 1, 3; Table 3). Interestingly, of the couples whose male partners reported perpetrating a form of IPV, between 25% and 60% of female partners disagreed that they had experienced that form of IPV (Table 3; Blocks 1–3; Column 1). Of the couples whose male partners reported perpetrating a form of IPV against their female partners while they were pregnant, between 50% and 85% of female partners disagreed that they had experienced that form of IPV while pregnant. Further, of the couples whose female partner reported experiencing a form of IPV, between 52–87% of men disagreed that they had perpetrated that form of IPV, and 90–97% disagreed that they had perpetrated that form of IPV during pregnancy (Table 3; Blocks 1–3; Column 3).

**Table 3:** Percentage of partner agreement in male and female partners' reports of IPV perpetration and victimization, by IPV type and timing, Tanzania, 2015.

		*n*	(1)		(2)		(3)		(4)	
			Couples in which men reported IPV perpetration and women reported no victimization		Couples in which men reported no IPV perpetration and women reported victimization		Couples in which women reported IPV victimization and men reported no perpetration		Couples in which women reported no IPV victimization and men reported perpetration	
			*n*	%	*n*	%	*n*	%	*n*	%
Block 1: Last 3 Mos	Physical IPV	450	69	59	381	31	147	81	303	14
	Sexual IPV	450	50	54	400	39	177	87	273	10
	Emotional IPV	450	127	35	323	53	253	68	197	23
	Economic IPV	450	115	60	335	32	153	70	297	23
Block 2: Last 12 Mos	Physical IPV	450	107	46	343	33	171	66	279	18
	Sexual IPV	450	59	49	391	40	187	84	263	11
	Emotional IPV	450	193	27	257	58	289	52	161	33
	Economic IPV	450	111	52	339	34	169	69	281	21
Block 3: Ever	Physical IPV	450	138	56	312	41	205	62	245	25
	Sexual IPV	450	67	49	383	47	213	84	237	14
	Emotional IPV	450	235	25	215	65	315	44	135	44
	Economic IPV	450	141	42	309	39	204	60	246	24
Block 4: Pregnancy	Physical IPV	432	2	50	430	8	35	97	397	0
	Sexual IPV	436	13	85	423	4	21	90	415	3
	Economic IPV	446	10	70	436	13	60	95	386	2

Finally, in Table 4, we reported, separately, estimates of chance-corrected agreement (*kappa*) between couples’ reports of men-to-women perpetration of each of the 48 acts of IPV considered in this analysis. The *kappa* estimates were low: for physical IPV acts, these estimates ranged between -0.01 and 0.03, 0.02 and 0.19 and 0.03 and 0.14 for last three months, twelve months and ever in the relationship, respectively; for sexual IPV acts, these estimates ranged between -0.02 and 0.06, 0.03 and 0.09, and 0.01 and 0.05 for last three months, twelve months and ever in the relationship, respectively; for emotional IPV acts, these estimates ranged between 0.01 and 0.07, 0.03 and 0.12, and -0.04 and 0.12 for last three months, twelve months and ever in the relationship, respectively; and, finally, for economic IPV acts, these estimates ranged between 0.05 and 0.14, 0.08 and 0.14, and 0.09 and 0.18 for last three months, twelve months and ever in the relationship, respectively.

**Table 4:** Couples agreement (Kappa) on intimate partner violence, n = 450 couples, Tanzania, 2015–2016.

		In last three months		In last twelve months		Ever in relationship		During pregnancy	
		*Kappa*	*p*	*Kappa*	*p*	*Kappa*	*p*	*Kappa*	*p*
Physical IPV	Slapped the woman or thrown something at her	0.01	0.45	0.10	0.02	0.10	0.01	—	—
	Pushed or shoved the woman	-0.02	0.72	0.02	0.30	0.03	0.22	—	—
	Hit the woman with a fist or with something else that could hurt her	-0.01	0.61	0.03	0.23	0.05	0.12	—	—
	Kicked, dragged, beaten, choked or burned the woman	0.03	0.14	0.14	0.00	0.14	0.00	—	—
	Threatened to use or actually used a gun, knife or other weapon against the woman	0.00	0.55	0.19	0.00	0.06	0.02	—	—
	Physical violence during pregnancy	—	—	—	—	—	—	0.05	0.01
Sexual IPV	Physically forced the woman to have sexual intercourse when she did not want to	0.06	0.04	0.09	0.01	0.05	0.10	—	—
	The woman had sexual intercourse when she did not want because she was afraid of what her partner might do	-0.02	0.79	0.03	0.15	0.01	0.27	—	—
	Sexual violence during pregnancy	—	—	—	—	—	—	0.08	0.04
Emotional IPV	Insulted a partner or deliberately make her feel bad about herself	0.06	0.08	0.09	0.03	0.10	0.02	—	—
	Belittled or humiliated the woman in front of other people	0.01	0.22	0.07	0.00	0.06	0.04	—	—
	Done things to scare or intimidate the woman on purpose	0.07	0.02	0.07	0.03	0.02	0.33	—	—
	Threatened to hurt the woman	0.05	0.08	0.03	0.25	-0.04	0.85	—	—
	Hurt people who the woman cared about	0.05	0.02	0.12	0.00	0.12	0.00	—	—
Economic IPV	Prohibited the woman from getting a job, going to work, trading or earning money	0.05	0.11	0.08	0.02	0.09	0.01	—	—
	Taken the woman’s money or earnings against her will	0.05	0.17	0.10	0.01	0.09	0.01	—	—
	Thrown the woman out of the house	0.09	0.03	0.08	0.04	0.18	0.00	—	—
	The man kept money from his earnings for alcohol, tobacco or other things for himself	0.14	0.00	0.14	0.00	0.13	0.00	—	—
	Economic violence during pregnancy	—	—	—	—	—	—	0.05	0.06

### Validity analysis results

Table 5 presents sample characteristics, and summary statistics of five IPV risk factors. The average man and woman were 41 and 36 years old, respectively. A vast majority of men (92%) and women (90%) had secondary or post-secondary level of schooling.

**Table 5:** Female and male partners' characteristics, n = 450 couples, Tanzania, 2015–2016.

	Mean	Std. Dev.
Female partners:		
Current age (in years)	35.98	10.60
Highest level of education attended (Ref: *Primary or none*)		
Secondary	0.77	0.42
Higher	0.13	0.34
Male partners:		
Current age (in years)	40.81	11.72
Highest level of education attended (Ref: *Primary or none*)		
Secondary	0.73	0.44
Higher	0.19	0.39
Attitudes on Gender Norms and Relations	44.20	6.54
Childhood trauma	19.01	3.55
Condom non-use (Ref: *condom use*)	0.72	0.45
Multiple sexual partners (Ref: *one sexual partner*)	0.28	0.45
Alcohol or drug use (Ref: *no*)	0.29	0.45

Table 6 shows prevalence of physical, sexual, emotional, economic, and any form of IPV against women in the last three months as reported by men, women, men or women, and men and women. Prevalence varied depending on whether men’s or women’s reports were used in the estimation. At least 50% more women than men reported occurrence of physical, sexual and economic IPV against women; 30% more women reported emotional IPV occurrences. Prevalence rates estimated using reports from men or women (Block 3) were 15–45% higher than those estimated using women’s reports (Block 2); 76–308% higher than those estimated using men’s (Block 1). Prevalence rates estimated using reports by men and women (Block 3) were 51–87% lower than those estimated using women’s reports (Block 2), and 27–60% lower than those estimated using men’s reports (Block 1).

**Table 6:** Prevalence of physical, sexual, economic, emotional or any form of violence in the last three months, as reported by men, women, either men or women, and both men and women, n = 450 couples, Tanzania, 2015–2016.

	Block 1		Block 2		Block 3		Block 4	
	Men's self-reports		Women's self-reports		Men's or women's self-reports		Men's and women's self-reports	
	Proportion	Std. Dev.	Proportion	Std. Dev.	Proportion	Std. Dev.	Proportion	Std. Dev.
Physical violence	0.15	0.36	0.33	0.47	0.42	0.49	0.06	0.24
Sexual violence	0.11	0.31	0.39	0.49	0.45	0.50	0.05	0.22
Emotional violence	0.26	0.44	0.34	0.47	0.49	0.50	0.10	0.30
Economic abuse	0.28	0.45	0.56	0.50	0.66	0.47	0.18	0.39
Any one form of violence	0.46	0.50	0.69	0.46	0.82	0.39	0.34	0.47

Table 7 shows for each IPV risk factor its estimated association with IPV against women as reported by men (Model 1), women (Model 2), and men and/or women (Models 3, 4). We expect that a valid indicator of IPV against women will have significant associations with most of the five IPV risk factors considered. All five risk factors were considered of equal significance as correlates of IPV against women in Tanzania.

**Table 7:** Multiple logistic regression analysis showing factors associated with men's IPV perpetration in last three months, n = 450 couples, Tanzania, 2015.

		Model 1			Model 2			Model 3			Model 4		
		Men's reports of IPV			Women's reports of men's IPV			Men's or women's reports of IPV			Men's and women's reports of IPV		
		OR	(s.e.)	*p*	OR	(s.e.)	*p*	OR	(s.e.)	*p*	OR	(s.e.)	*p*
Panel 1: Physical IPV	Men's inequitable gender attitudes	1.03	(0.03)		1.05	(0.02)	*	1.05	(0.01)	**	1.05	(0.04)	
	Men's traumatic childhood	1.10	(0.05)	*	1.08	(0.03)	**	1.09	(0.03)	**	1.16	(0.05)	**
	Men's condom non-use (*yes* = 1)	0.55	(0.16)	*	0.92	(0.18)		0.60	(0.08)	***	1.35	(0.68)	
	Men's multiple sexual partners (*yes* = 1)	1.19	(0.25)		0.90	(0.28)		1.00	(0.26)		1.11	(0.41)	
	Men's alcohol or drug use (*yes* = 1)	2.01	(0.73)	*	1.65	(0.29)	**	1.84	(0.42)	**	2.67	(0.85)	**
Panel 2: Sexual IPV	Men's inequitable gender attitudes	1.00	(0.02)		1.02	(0.02)		1.01	(0.01)		1.03	(0.05)	
	Men's traumatic childhood	1.11	(0.07)		1.02	(0.03)		1.05	(0.03)		1.11	(0.04)	**
	Men's condom non-use (*yes* = 1)	0.77	(0.44)		1.07	(0.27)		1.11	(0.29)		0.54	(0.19)	
	Men's multiple sexual partners (*yes* = 1)	3.06	(0.79)	***	0.90	(0.27)		1.32	(0.40)		1.94	(0.99)	
	Men's alcohol or drug use (*yes* = 1)	1.56	(0.23)	***	0.86	(0.13)		0.98	(0.18)		1.30	(0.42)	
Panel 3: Emotional IPV	Men's inequitable gender attitudes	1.02	(0.01)		1.01	(0.03)		1.01	(0.03)		1.03	(0.01)	*
	Men's traumatic childhood	1.18	(0.06)	**	1.05	(0.03)		1.07	(0.04)	*	1.19	(0.05)	***
	Men's condom non-use (*yes* = 1)	0.56	(0.11)	**	0.89	(0.21)		0.69	(0.12)	*	0.63	(0.18)	
	Men's multiple sexual partners (*yes* = 1)	1.14	(0.22)		0.79	(0.14)		0.99	(0.19)		0.84	(0.11)	
	Men's alcohol or drug use (*yes* = 1)	2.77	(0.48)	***	1.62	(0.24)	***	2.70	(0.44)	***	2.13	(0.54)	**
Panel 4: Economic IPV	Men's inequitable gender attitudes	1.06	(0.02)	***	1.01	(0.02)		1.05	(0.02)	**	1.01	(0.02)	
	Men's traumatic childhood	1.12	(0.04)	***	1.04	(0.04)		1.08	(0.05)		1.12	(0.05)	*
	Men's condom non-use (*yes* = 1)	0.54	(0.13)	*	0.95	(0.24)		0.82	(0.15)		0.47	(0.20)	
	Men's multiple sexual partners (*yes* = 1)	1.29	(0.29)		1.01	(0.29)		1.17	(0.32)		1.15	(0.35)	
	Men's alcohol or drug use (*yes* = 1)	2.20	(0.57)	**	1.44	(0.24)	*	1.63	(0.36)	*	3.14	(1.12)	***
Panel 5: Any IPV	Men's inequitable gender attitudes	1.05	(0.02)	**	1.01	(0.02)		1.02	(0.02)		1.05	(0.02)	**
	Men's traumatic childhood	1.16	(0.05)	***	1.06	(0.03)		1.06	(0.06)		1.18	(0.05)	***
	Men's condom non-use (*yes* = 1)	0.50	(0.14)	*	0.83	(0.22)		0.44	(0.16)	*	0.68	(0.19)	
	Men's multiple sexual partners (*yes* = 1)	1.63	(0.20)	***	0.68	(0.15)		1.19	(0.41)		1.00	(0.21)	
	Men's alcohol or drug use (*yes* = 1)	2.56	(0.62)	***	1.20	(0.30)		1.93	(0.52)	*	2.05	(0.37)	***

***p≤.001

**p≤.01

*p≤.05.

All models are adjusted for men's and women's highest level of schooling attended and age. Standard errors are adjusted for clustering of data at village level.

Of five IPV risk factors, three (childhood trauma; condom non-use; alcohol or drug use) had significant associations with men’s self-reported physical IPV perpetration in the last three months (Panel 1; Model 1); and, three (gender inequitable attitudes; condom non-use; alcohol or drug use) had associations with women’s self-reported victimization (Panel 1: Model 2). The magnitudes of association were relatively small; directions generally were as expected. Men’s odds (aOR = 1.10) of reporting perpetration increased with a unit increase in their childhood-trauma scores. Alcohol or drug users had higher odds (aOR = 2.01) than non-users; condom non-users (aOR = 0.55) had lower odds than users. Next, men who reported agreement with more gender-inequitable attitudes had higher odds (aOR = 1.05) of being reported as perpetrators by their female partners. The same pattern of association was evident for men who experienced more trauma during childhood (aOR = 1.08). Finally, alcohol or drug users had higher odds (aOR = 1.65) of being reported as perpetrators compared to non-users.

Of five IPV risk factors, two (multiple sexual partners; alcohol or drug use) had significant associations with men’s self-reported sexual IPV perpetration (Panel 2; Model 1); none had associations with women’s self-reported victimization (Panel 2: Model 2). Men who reported multiple sexual partners were three times more likely to report sexual IPV perpetration than men who reported one or none. Men who reported alcohol or drug use had higher odds (aOR = 1.56) than men who reported non-use.

On emotional IPV (Panel 3), three (childhood trauma; condom non-use; alcohol or drug use) of five IPV risk factors had significant associations with men’s self-reported perpetration (Model 1); one (alcohol or drug use) had an association with women’s self-reported victimization (Model 2). Men who reported more traumatic childhood had higher odds (aOR = 1.18) of reporting perpetration. Condom non-users had lower odds (aOR = 0.56) than users; alcohol or drug users had higher odds (aOR = 2.77) than non-users. Further, alcohol or drug users had higher odds (aOR = 1.62) of being reported as perpetrators by women than non-users.

On economic IPV (Panel 4), four (inequitable gender attitudes; childhood trauma; condom non-use; alcohol or drug use) of five IPV risk factors, had significant associations with men’s self-reported perpetration (Model 1); one (alcohol or drug use) was associated with women’s self-reported victimization (Model 2). Men who reported agreement with more gender-inequitable attitudes (aOR = 1.06) and more childhood-trauma (aOR = 1.12) had higher odds of reporting perpetration. Condom non-users had lower odds (OR = 0.54) than users; alcohol or drug users had had higher odds (OR = 2.20) than non-users. Further, alcohol or drug users had higher odds (aOR = 1.44) of being reported as perpetrators by their female partners.

All five IPV risk factors (Panel 5) had significant associations with men’s odds of reporting of physical, sexual, emotional or economic IPV perpetration in the last three months (Model 1). None had associations with women’s odds of reporting victimization (Model 2).

Further, we estimated associations with five IPV risk factors of IPV occurrences reported by men or women (Model 3) and by both men and women (Model 4). Of five IPV risk factors, four (inequitable gender attitudes; childhood trauma; condom non-use; alcohol or drug use) were associated with physical IPV, none with sexual IPV, three with emotional IPV (childhood trauma; condom non-use; alcohol or drug use), two each with economic IPV (inequitable gender attitudes; alcohol or drug use) and any one form of IPV (condom non-use; alcohol or drug use), when IPV against women was measured using IPV occurrences reported by men or women (Model 3). Additionally, of five IPV risk factors, two (childhood trauma; alcohol or drug use) were associated with physical IPV, one (childhood trauma) with sexual IPV, three (inequitable gender attitudes; childhood trauma; alcohol or drug use) with emotional IPV, two (childhood trauma; alcohol or drug use) with economic IPV, and three (inequitable gender attitudes; childhood trauma; alcohol or drug use) with any one form of IPV, when IPV against women was measured using IPV occurrences reported by both men and women (Model 4).

## VIII. Discussion and conclusions

In recent years, the Government of Tanzania has amplified its effort to redress the consequences faced by the victims of intimate partner violence in the country. These efforts, albeit important, may become those of limited impact if assessment tools are neither specific nor sensitive in correctly classifying women as victims or otherwise. Self-reports by the victim are commonly used for IPV assessment. Yet, self-reports are prone to reporting bias. The validity of self-reports by the victim is contingent on victims recognizing an act as violent as and when it happens, remembering when violence occurs in the relationship, and not concealing violence. Self-reports by the victim and the perpetrator are believed to mitigate reporting biases (e.g., underreporting).

In this paper, using data from 450 Tanzania couples, we examined the level of inter-partner agreement in the reporting of physical, sexual, emotional, and economic IPV against women in the last three months prior to the survey, last twelve months, ever in the relationship, and during pregnancy. Further, we examined the relative association of men’s self-reported perpetration and women’s self-reported victimization with validated risk factors, namely, men’s gender inequitable attitudes, exposure to childhood trauma, condom non-use, multiple sexual partners, and alcohol or drug use. We used men’s self-reports to measure risk factors.

In IPV reporting, we found poor agreement between partners within the couple in Northern Tanzania. We found that, for every violence type across the recall periods of last three months, last twelve months and ever in the relationship, at least one in three couples disagreed about IPV occurrences in the relationship. The level of partner disagreement in the reporting of lifetime physical IPV in the current analysis (42%) is comparable to findings (35%) by Yount and Li (2012) using the 1995–96 and 1996–97 DHS data from 943 randomly selected couples from Assiut and Souhag, Egypt [11].

We argue that, in the context of Northern Tanzania, definitional differences have contributed to discordant reporting more than norms of victim blaming, social desirability, or recall bias. Compared to women LIMCA members trained in child protection and gender-based violence, men may be less capable of recognizing acts as those of violence due to training gaps, potentially resulting from men’s non-participation in any structured IPV training. That the level of couple disagreement is higher in the reporting of emotional violence (48%) than it is in the reporting of physical violence (36%) provides some *prima facie* support to our argument. Norms of victim blaming should similarly affect IPV reporting by women LIMCA members and their male partners, and cannot explain discordant reporting. Social desirability bias is less likely to be a factor: gendered motivation to conceal or disclose IPV occurrences are likely slim in the gender-stratified context of Northern Tanzania, where IPV is perceived as normal and met with acceptance by both genders. That the extent of couple disagreement remains comparable across the recall periods of last three months, last twelve months is suggestive of the fact that men and women were equally prone to recall bias.

A vast majority of couples agreed that physical (92%), sexual (93%), and economic (85%) abuse did not occur during pregnancy. Such high concordance rates were contrary to expectation, given language ambiguity in the questions of interest. These questions do not ask about abuse in reference to a specific pregnancy, requiring each partner in the couple of multiple pregnancies to decide for him/herself which pregnancy to recall in his/her answer. Specifying pregnancies in questions about abuse during pregnancy will further improve data quality.

Convergent validity findings via logistic regression models with village-level clustered errors were somewhat contrary to expectation. Compared to women’s self-reports, men’s had significant associations with at least as many IPV risk factors. This implies that men’s self-reports are as valid as women’s self-reports as an indicator of men’s perpetration of physical IPV against women, and more valid than women’s self-reports as an indicator of men’s perpetration of sexual, emotional, and economic IPV in the context of Northern Tanzania.

Further, validation findings suggest that some women may have reported sexual, emotional, or economic IPV when there were none. Over-reporting (i.e., “the endorsement of survey items about aggressive acts under conditions where neutral third parties would not have considered the event as IPV” [71]) may stem from motivations including: “wanting one partner to be seen in an unfavorable light, getting revenge for relationship conflicts, needing to feel superior to one’s partner” [14]. Otherwise, over-reporting may happen in hopes of economic or social benefits (i.e., perverse incentive). Since, to our knowledge, no such benefits were directed at the sampled women at the time of the interview, women’s IPV reporting behavior is puzzling, requiring further scrutiny.

The validation analysis results should be interpreted in light of the limitations as follows. The sample of 450 couples was not randomly selected, suggesting current results may or may not be generalizable to all couples whose female partners are engaged in income-generating activities in Tanzania. Further, more importantly, we used men’s self-reports to measure all five risk factors for IPV perpetration in men. Therefore, the unbiasedness of validation results are conditional on five risk factors being: (a) true risks in the context of Tanzania; and, (b) free of measurement errors. Future studies should replicate current analyses using different data.

In conclusion, the use of retrospective self-report questionnaires has revolutionized the way we collect data on violence perpetration and victimization, allowing production of a great deal of knowledge about the causes and consequences of interpersonal violence against women. However, the lone use of retrospective self-reports is believed to have created controversies (e.g., gender differences in self-reports of intimate partner violence). Research attempting to resolve these controversies, as part of research on the operationalization of IPV, is more prevalent in high- vs. low-income countries. We recommend this gap be addressed urgently. In the meantime, researchers and practitioners may modify current data-collection practices in low-income countries. For instance, in addition to collecting data on IPV occurrence, researchers and practitioners should collect data on factors affecting the quality of IPV reports and reporting. These factors include: satisfaction level, a desire to exact revenge for prior mistreatment, the degree of investment in the relationship, among other relationship factors. Valid assessment is imperative to mitigating IPV adversities on maternal and child health.

## Supporting information

S1 Dataset PLOS One Data Sharing 20DEC2017.zip. (ZIP)
